# Recognition of Banknote Fitness Based on a Fuzzy System Using Visible Light Reflection and Near-infrared Light Transmission Images

**DOI:** 10.3390/s16060863

**Published:** 2016-06-11

**Authors:** Seung Yong Kwon, Tuyen Danh Pham, Kang Ryoung Park, Dae Sik Jeong, Sungsoo Yoon

**Affiliations:** 1 Division of Electronics and Electrical Engineering, Dongguk University, 30 Pildong-ro 1-gil, Jung-gu, Seoul 100-715, Korea; sbaru07@dgu.edu (S.Y.K.); phamdanhtuyen@gmail.com (T.D.P.); jungsoft97@dongguk.edu (D.S.J.); 2 Kisan Electronics, Sungsoo 2-ga 3-dong, Sungdong-gu, Seoul 133-831, Korea; ssyoon@kisane.com

**Keywords:** fitness classification, contact image sensor, fuzzy system, USD, KRW, Indian rupee (INR)

## Abstract

Fitness classification is a technique to assess the quality of banknotes in order to determine whether they are usable. Banknote classification techniques are useful in preventing problems that arise from the circulation of substandard banknotes (such as recognition failures, or bill jams in automated teller machines (ATMs) or bank counting machines). By and large, fitness classification continues to be carried out by humans, and this can cause the problem of varying fitness classifications for the same bill by different evaluators, and requires a lot of time. To address these problems, this study proposes a fuzzy system-based method that can reduce the processing time needed for fitness classification, and can determine the fitness of banknotes through an objective, systematic method rather than subjective judgment. Our algorithm was an implementation to actual banknote counting machine. Based on the results of tests on 3856 banknotes in United States currency (USD), 3956 in Korean currency (KRW), and 2300 banknotes in Indian currency (INR) using visible light reflection (VR) and near-infrared light transmission (NIRT) imaging, the proposed method was found to yield higher accuracy than prevalent banknote fitness classification methods. Moreover, it was confirmed that the proposed algorithm can operate in real time, not only in a normal PC environment, but also in an embedded system environment of a banknote counting machine.

## 1. Introduction

Problems occur when banknotes used in such devices as automated teller machines (ATMs) and vending machines cannot be recognized due to higher-than-normal levels of soilage. This can cause these devices to malfunction, force users to re-insert the banknotes, or create other inconveniences during the insertion of the banknotes. Banknote fitness classification calculates the quality of a banknote to determine whether it is usable. Incorrect fitness classification can lead to unusable banknotes being circulated again or usable banknotes being discarded, which is also problematic because of the cost of discarding bad banknotes and creating new ones. Previous researches have thus been conducted on fitness classification because of growing demand for technology that can accurately perform such classification.

In research by the De Nederlandsche Bank on efficient banknote usage, using banknote data acquired via color imaging [1,2], soiling was found to be one of the main reasons for the decline in the fitness of banknotes with use over time [1]. Other research has also involved the use of color images of banknotes to extract soil characteristics [3,4,5]. Geusebroek *et al.* [1] and Balke *et al.* [5] proposed a machine learning technique for fitness classification on Euro banknotes using the mean and the standard deviation of brightness values extracted from square-shaped areas in banknote images. Intensity, red-green-blue (RGB) color, and a mix of the yellow-blue and red-green channel colors were used as fitness classification features. The classifiers used in [1,5] were a mixture of simple linear weak classifiers using an adaptive boosting (AdaBoost) algorithm. Instead of proposing an automatic fitness classification method, Balke *et al.* described the classes of, and various contributing factors in, fit banknotes and unfit banknotes [3]. The research in [4] did not propose an automatic fitness classification method, but instead presented test values to classify the levels of soiling of Euro banknotes into five categories using various sensors [4]. Aoba *et al.* proposed a technique for recognizing Euro banknote denomination and fitness classification using visible light reflection images and near-infrared light reflection images as input data [6]. This research involved a classification part, which used a three-layered perceptron, and a verification part, which used a radial-based function (RBF) network to reject partial unfit data. He *et al.* performed research that classified the fitness of Chinese (Renminbi (RMB)) banknotes using a neural network [7]. In this technique, the gray level histogram of the image of a banknote was used as an input feature, and the classifier was designed based on a neural network that used a sine basis function [7].

Pham *et al.* captured Indian banknote images with a visible light camera, performed a discrete wavelet transform (DWT) on pre-selected regions of interest (ROI), and used a support vector machine (SVM) to classify the banknotes’ fitness using the mean and the standard deviation of image brightness selected from the transformed banknotes based on a correlation with densitometer data [8]. Rather than finding fitness levels through banknote images, Kang *et al.* [9] and Teranishi *et al.* [10] performed fitness classification by using acoustic energy, which is emitted when a banknote is inserted into a counting machine and passes over the roller.

Most of the methods proposed in relevant research require the training procedures for fitness classification. A fairly large amount of data and a lot of time are required for such training. Moreover, if there is a change in the national currency being used for classification, difficulties arise because the training procedure would need to be performed again, and new data relating to the currency would need to be gathered.

To solve problems in prevalent technologies for banknote fitness classification, this study proposes a method that uses a fuzzy system to achieve accurate fitness classification results from visible light and infrared images using rules determined. There exist various factors such as crumpling and tear, etc, which affect the banknote fitness, and our research is focused on the factor of soiling level on banknote surface for the classification of banknote fitness.

In comparison with previous researches, the results presented herein are novel in the following four ways:

- First, this is the first study to carry out fitness classification of all denominations of the United States Dollar (USD) and the Korean Won (KRW).
- Second, the regions of interest (ROIs) for extracting features from the visible light reflection (VR) images and near-infrared light transmission (NIRT) images were set differently according to banknote denomination and direction; from these ROI, the average values of the VR and NIRT images were extracted as features.
- Third, a fuzzy system was applied to the features acquired from input images to determine the final fitness values for the input images and determine whether the relevant banknotes were usable based on two classes (Fit, Unfit).
- Fourth, by using a fuzzy system, high performance of fitness classification can be achieved regardless of the currency of the banknote used, such as USD, KRW, and INR.

The remainder of this paper is structured as follows: Section 2 contains a description of the proposed algorithm for banknote fitness classification. Section 3 is devoted to a presentation of the results of experimental performance analyses of the algorithm, and Section 4 summarizes the conclusions of this study as well as plans for future research in the area.

## 2. Proposed Method

### 2.1. Overview

**Figure 1:**
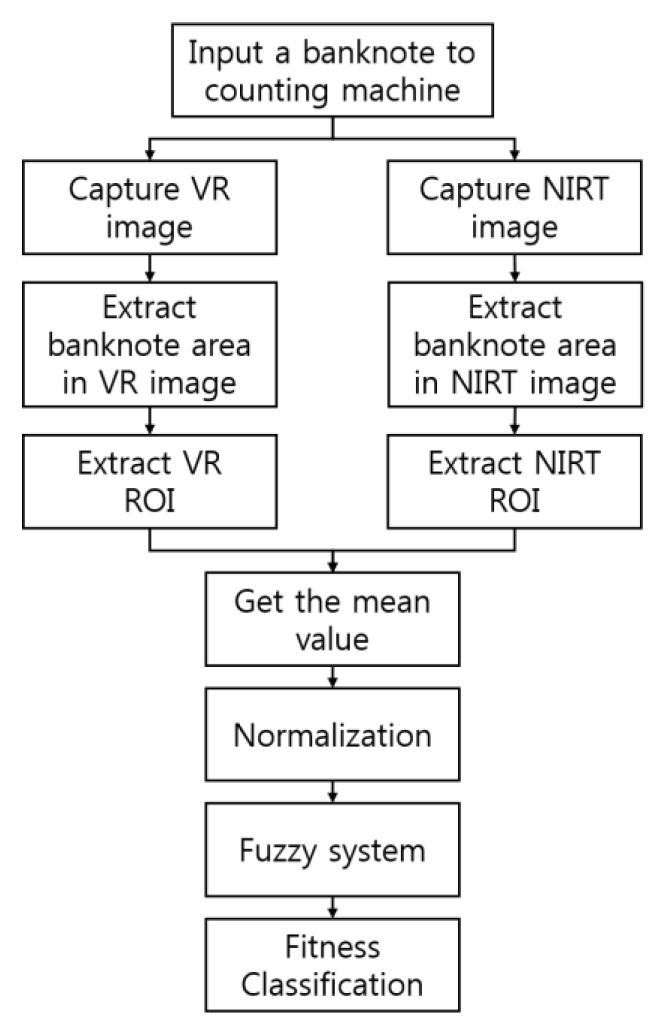
Flowchart of proposed method.

**Figure 2:**
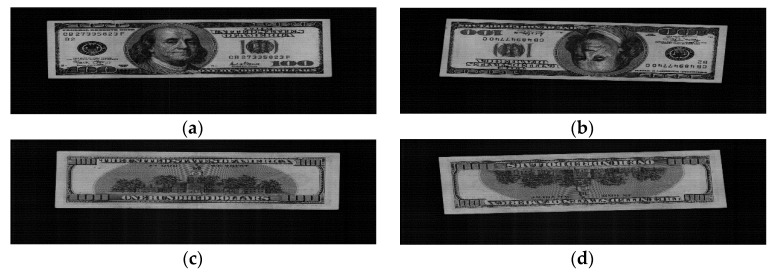
Examples of images of a banknote captured in four directions: (**a**) A; (**b**) B; (**c**) C; (**d**) D.

Figure 1 shows a flowchart of the proposed method. When a banknote is inserted into a counting machine, images of the banknote can be captured from four directions (A, B, C, and D), as shown in Figure 2. Here, direction A shows an image of the banknote in the front side and forward direction, direction B shows an image in the front side and backward direction. C represents an image in the back side and forward direction. Direction D shows the image of the banknote in the back side and backward direction.

The banknote area is detected such that the background is excluded from the captured banknote image, and ROI within this area are selected from each VR image and NIRT image according to denomination. The average pixel value for each ROI is captured as well.

To use the captured features as input values in a fuzzy system, normalization is carried out by using a min-max scaling of each mean value to a value between 0 and 1. The two values are used as input to determine fuzzy rules followed in the fuzzification and defuzzification processes conducted to create a single value that can classify the degree of soil on the banknote. At the fitness classification step, the value produced as output from the fuzzy system determines whether the entered banknote is fit according to a preset threshold value.

### 2.2. Image Acquisition and Feature Extraction

In order to perform banknote fitness classification, we need to understand how the features of a banknote usually change as new money becomes worn money. As a banknote is used, its color fades, and its light reflectivity and transmission rate decrease due to substances sticking to it rather than wear and tear [3]. Therefore, in this study, features for fitness classification were selected with these characteristics in mind. As a banknote becomes older, dust and other particles combine with oils from people’s skin to form uneven layers that lower the reflection rate. The oils also increase the absorption rate of the banknote, which lowers the transmission rate of light through it.

Newly issued banknotes are formed by treating the original sheet (the main part of the face of a banknote) with adhesive material and printing on the surface with ink. As a banknote is used, its surface becomes rougher, and it is soiled with various materials. Normally, the soiling material is composed of people’s sweat, oils, dust, *etc.* Moreover, the thickness of the banknote decreases over time. Light that shines on the banknote reflects or transmits. The surface of new money is relatively smooth, due to which there is a mirror reflection effect; on the contrary, the rough surface of old money is irregular, relatively severer than that of new money, because of which there is a diffuse reflection effect that scatters the light that shines on it. Therefore, reflected energy from any given direction can be expected to be less in the case of old money than new money due to diffusive reflection. In the case of transmission, people’s sweat, oils, dust, *etc.*, stuck to banknotes absorb light energy, which normally reduces transmission rate. Even though surface abrasion reduces the thickness of banknotes as well as absorption, the path of the transmitted light diffuses, and light energy received by a sensor from the relevant direction decreases accordingly. To summarize the light reflection and transmission mechanisms in banknotes thus far, reflection is expected to be reduced in old money due to surface roughness, and transmission is expected to be reduced in old money due to energy absorption by soiling materials [3].

Based on these characteristics, VR and NIRT images are used as features for fitness detection in this study. If only VR images were used, fitness classification would need to be performed using only the amount of reflected visible light illumination, and if only one side of the banknote was dirty, this would pose a problem because fitness classification results for the same banknote would vary depending the side captured. To overcome these issues, both VR images and NIRT images are used as features for fitness classification.

**Figure 3:**
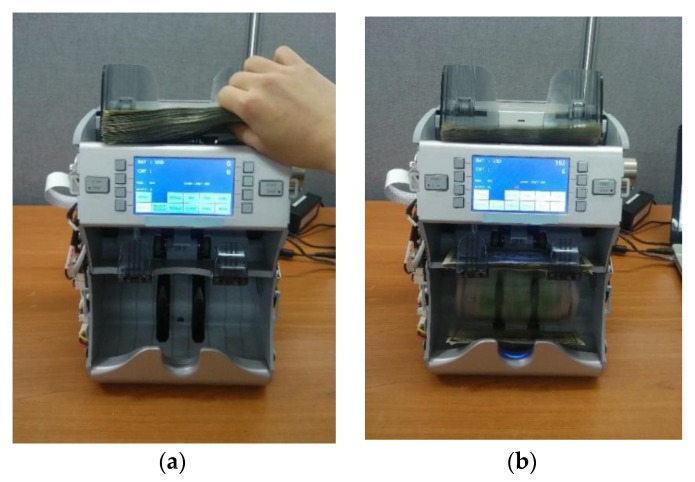
Example of capturing images of banknotes: (**a**) Inserting banknotes; (**b**) Banknote image capture.

To capture banknote images, a commercial banknote counting machine was used in this study [11]. Figure 3 shows an example of banknote images being captured for this research. As can be seen in Figure 3a, the banknotes were inserted into the banknote counting machine; as Figure 3b shows, the VR images and the NIRT images were captured automatically as the banknotes passed through the banknote counting machine.

Because of the limited size and cost of the banknote counting machine, the images were captured using a one-dimensional line contact image sensor (CIS) rather than a two-dimensional area sensor, which is used in traditional digital cameras. Therefore, rather than an image of the entire area being captured at once, the image was captured sequentially, one line at a time. The banknote inserted into the banknote counting machine was moved by high-speed rollers (over 800 ppm (pulses per minute)), as a light-emitting diode (LED) and near-infrared LED illumination were used alternately to capture the image with one line (by the CIS) at a time. There are 1584 pixels on the one-dimensional line CIS. For the VR image, a 350-line image was captured, whereas for the NIRT image, a 175-line image was captured. That is, for the VR image, the 350 captured line images were sequentially composed into a final 2D banknote image with 1584 × 350 pixels, whereas for the NIRT image, the 175 captured line images were sequentially composed into a final 2D banknote image with 1584 × 175 pixels. Compared to the width (1584 pixels) of the image (where the width is determined by 1584 pixels on the one-dimensional line CIS), the height (350 or 175 pixels) of the image is much small. Therefore, all the images of Figure 2 look elongated, but they are the original captured images which are not arbitrarily elongated.

**Figure 4:**
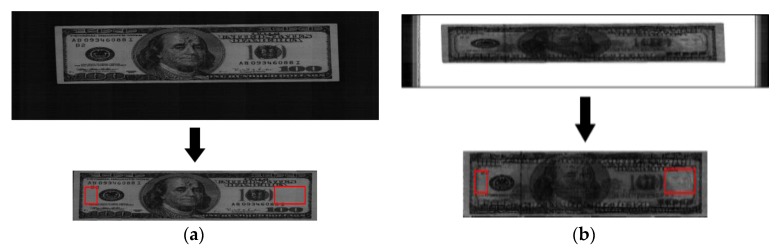
The banknote area extracted from the banknote image and the regions of interest: (**a**) VR image; (**b**) NIRT image.

A corner detection algorithm installed in the banknote counting machine was used to extract the banknote area from the captured banknote images. The image of extracted banknote area can be seen in the second row of Figure 4.

As mentioned above, banknotes inserted in the banknote counting machine normally have four directions (A, B, C, and D). The reason for this division of banknote directions is that the brightness values of areas with the least number of markings (ROI) in the VR and the NIRT images are extracted as features to use for fitness classification, and ROI from which features are extracted differ depending on the banknote’s denomination and direction. The commercial banknote counting machine used in this research included the functionality of classifying denomination and direction [11]; by using this, features were extracted for fitness classification from different ROIs specified according to denomination and direction.

As shown in Figure 4a, the banknote area was first extracted from the original VR image, and the specified ROI in the extracted banknote image are shown as red-colored boxes. In Figure 4b, the banknote area was extracted from the original NIRT image, and the specified ROI in the extracted banknote image are shown as red-colored boxes.

The average pixel values from each of the ROIs in the VR image and the NIRT image were used as the two feature values in this research. To use the captured features as input in the fuzzy system, each average value was normalized through min-max scaling to a value between 0 and 1.

This normalization process was needed because the features from VR and NIRT images recorded different values and ranges. Furthermore, the ranges of feature value of the VR image and the NIRT image can change irregularly depending on the degree of banknote soiling. For these reasons, each feature value was normalized to a value between 0 and 1 for this study. These two values were used as input to specify fuzzy rules followed in fuzzification and defuzzification processes to create a single value to specify the degree of soiling on the currency.

The min and max values were decided from the mean values of ROIs of experimental banknote images. In our system, the average of pixel values in ROI can be varied by temperature. In order to solve this problem, we use the following scheme. As shown in Figure 2, the input captured image includes both the banknote area and dark background. Through the procedure of extracting banknote area as shown in Figure 1, the banknote area can be separated from dark background. Then, our system measures the average pixel value of dark background, and all the pixel values in ROI are compensated by the way of setting the average pixel value of dark background to be zero. For example, if the average pixel value of dark background in input image is calculated as 8, all the pixel values in ROI are reduced by 8. This is because if the pixel values of input image are changed by temperature, those of dark background are also changed. Consequently, based on the average pixel value of dark background, all the pixel values in ROI is compensated, which can reduce the effect by the change of temperature. Then, these two normalized values were used as inputs to fuzzy system, and based on the single output score (representing the degree of soiling on the currency) of fuzzy system, final decision of fitness or unfitness of input banknote was made.

### 2.3. Fitness Classification Using a Fuzzy System

What follows is a detailed description of the fuzzy system used in this research. Table 1 shows the fuzzy rule table designed for and used in this research. There are three input values: “L (Low)”, “M (Medium)”, and “H (High)”. There can be two output values: “L (Low)” and “H (High)”. For example, if the magnitude of the feature value of the VR image (VR mean) as well as that of the NIRT image (NIRT mean) is small, the relevant banknote’s degree of cleanliness can be considered low. That is, if the feature value of the VR image is “L” and that of the NIRT image is “L”, the banknote’s fitness classification value can be expected to be “L”. The fuzzy rule table shown in Table 1 was designed based on these characteristics. Normally in fuzzy logic, if two inputs are used, an IF-THEN rule is used [12]. In this study, the two feature values (VR and NIRT) were both considered at the same time to perform the final fitness classification; hence, among the IF-THEN rules of AND and OR operations, an AND operation was used.

**Figure 5:**
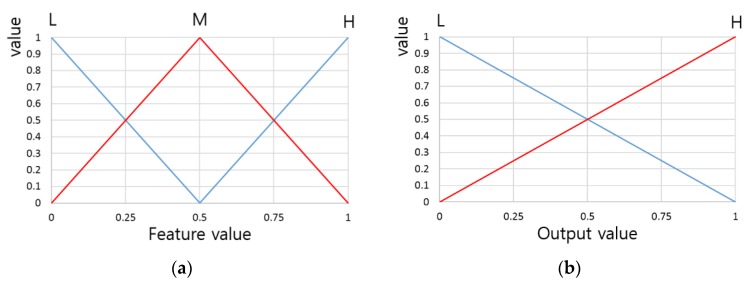
Banknote fitness classification system membership functions: (**a**) Input membership function; (**b**) Output membership function.

In this research, a linear (or triangular) fuzzy membership function was used, as shown in Figure 5. That is because due to the advantages of high computation speed and low code complexity, this function is the most commonly used one [13,14,15]. According to Table 1, there are three input membership functions—“L (Low)”, “M (Medium)”, and “H (High)”—as shown in Figure 5a, and two output membership functions—“L (Low)” and “H (High)”—as shown in Figure 5b.

In this research, two features (VR mean and NIRT mean) were used, each of which could have three values (L, M, H); thus, there were a total of nine input value pairs ((L, L), (L, M), (L, H), (M, L), (M, M), (M, H), (H, L), (H, M), (H, H)), and a single output value was determined from these. When determining the output value, we can use the Max rule method, which selects the largest one of the two input values, or the Min rule method, which selects the smallest [15].

**Figure 6:**
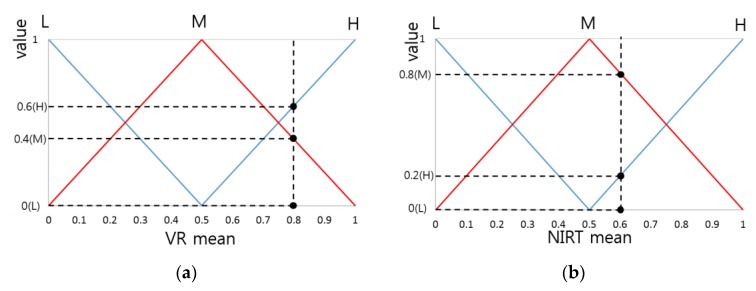
Example of calculating output value from input feature value input membership function: (**a**) Output value for VR mean; (**b**) Output value for NIRT mean.

For example, through the input membership function, a VR mean value of 0.8 has output values of 0 (L), 0.4 (M), and 0.6 (H), as shown in Figure 6. Furthermore, if the mean value of NIRT is 0.6, it has output values of 0 (L), 0.8 (M), and 0.2 (H). Based on these two types of output values, nine combinations can be found: (0(L), 0(L)), (0(L), 0.8(M)), (0(L), 0.2(H)), (0.4(M), 0(L)), (0.4(M), 0.8(M)), (0.4(M), 0.2(H)), (0.6(H), 0(L)), (0.6(H), 0.8(M)), (0.6(H), 0.2(H)). If there is a combination of (0(L), 0.8(M)), the value of 0 can be selected through the Min rule. Moreover, by looking at Table 1, if the VR mean is L and the NIRT mean is M, the output value is L, so that L can be selected through the fuzzy rules. That is, through the Min rule and the fuzzy rule, an inference value (IV) of 0(L) can be obtained from (0(L), 0.8(M)). If the Max rule is used, an IV of 0.8(L) can be obtained. Since there is a total of nine combinations, there are nine IVs. Table 2 shows examples of the IVs that can be obtained from the combinations through the Min rule, the Max rule, and the fuzzy rule.

**Figure 7:**
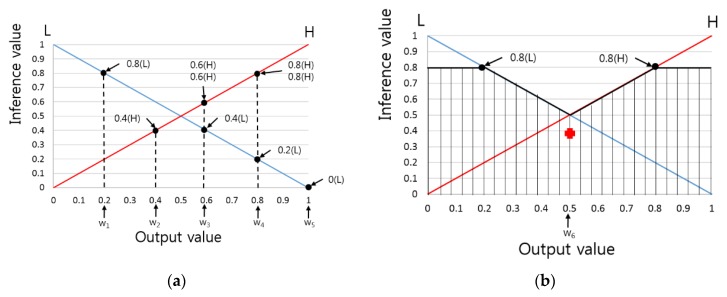
Examples of output values found when the IVs were used as input for various defuzzification methods: (**a**) FOM, LOM, MOM, MeOM; and (**b**) COG.

As shown in Figure 7a, a single IV can be used to obtain several output values. For example, an IV of 0.8(L) can yield output value w_1_. Defuzzification can be used to produce a single final output value from these multiple values, and this output value is used as the expected fitness classification value of the banknote. Among a variety of prevalent defuzzification methods commonly used, the performance of six methods was compared: First of Maxima (FOM), Last of Maxima (LOM), Middle of Maxima (MOM), Mean of Maxima (MeOM), Center of Gravity (COG), and Revised Weighted Average (RWA) [16,17].

The FOM, LOM, MOM, and MeOM respectively select the first, last, middle, and mean of the output values calculated by the maximum value among the obtained IVs. COG finds an output value that is the center of gravity of a polygon (marked with stripes of Figure 7b) formed from the maximum values of the obtained IVs. In Figure 7a,b, the final output values obtained from each method are w_1_ for FOM, w_4_ for LOM, ((w_1_ + w_4_)/2) for MOM, ((w_1_ + w_4_ + w_4_)/3) for MeOM, and w_6_ for COG. RWA uses a weighted average, and is similar to COG in that it finds the mean area, but different in that RWA finds the mean after multiplying the output value calculated from all IVs by the center of gravity measure of the output membership function. 

A single score was created from the defuzzification methods shown in Figure 7, and banknote fitness classification was performed using this score. A threshold value was determined according to the banknote’s denomination and direction; if the output score was higher than the threshold, the banknote was classified as fit, in that it could be used; however, if not, the banknote was classified as an unfit banknote, and hence could no longer be used.

## 3. Experimental Results

For the first experiment, we used the US dollar database to evaluate the performance of the proposed fitness classification method. This database contained images of denomination of $1, $2, $5, $10, $20, $50, and $100 banknotes, each of which was captured from four directions as shown in Figure 2. The number of banknote images used in this research is shown in Table 3. 

Based on previous research that presented actual densitometer measurement values [18] and those based on soiling levels, human experts classified the banknotes in the database as fit banknotes (capable of being used) or unfit banknotes (incapable of use) as shown in Table 4.

**Figure 8:**
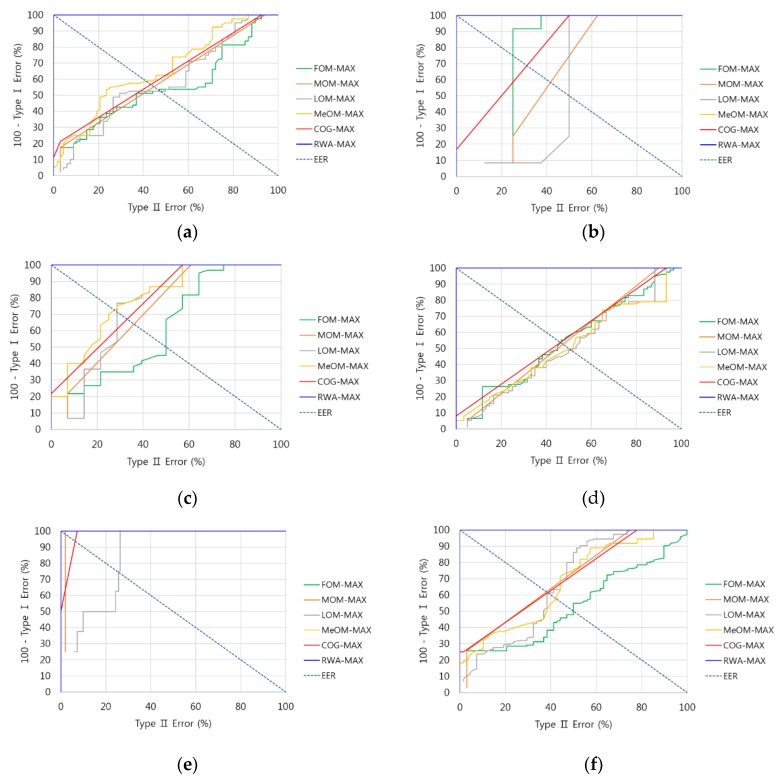

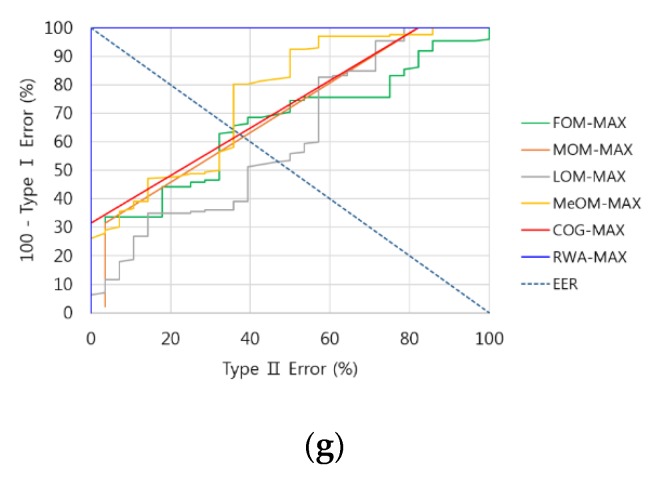
Examples of ROC curves for the error in each defuzzification method when using the Max rule: (**a**) $1 direction A; (**b**) $2 direction A; (**c**) $5 direction A; (**d**) $10 direction A; (**e**) $20 direction A; (**f**) $50 direction A; (**g**) $100 direction A.

In this study, Min and Max rules as well as six defuzzification methods (FOM, LOM, MOM, MeOM, COG, and RWA) were used to test performance. We defined two types of errors: Type I and Type II errors. A Type I error occurred when a fit banknote was incorrectly classified as unfit, and a Type II error occurred when an unfit banknote was incorrectly classified as fit. As explained at the end of Section 2.3, depending on the threshold of the output of the fuzzy system, Type I and Type II errors were switched. Based on the threshold, which existed when the difference between Type I and Type II errors was minimized, the equal error rate (EER) was found, which is the mean error of Type I and Type II errors. The experimental results showed that when the Max rule and the RAW method were used for fitness classification, the EER was at its lowest at 0.04%. Figure 8 shows examples of receiver operational characteristic (ROC) curves for errors according to each defuzzification method when the Max rule was used in direction A data for each denomination. As shown in Figure 8, when the RWA and the Max rule were used, the error rate was lowest.

The RWA method recorded a higher degree of accuracy than the COG method in fitness classification. This is because in case of COG, the center of gravity was calculated for the area under the maximum value of the L and H output membership functions among the IVs. However, for the output membership function used in this research, the shape exhibited left-right symmetry as shown in Figure 5b; thus, the area’s center of gravity tended to cluster around 0.5.

**Figure 9:**
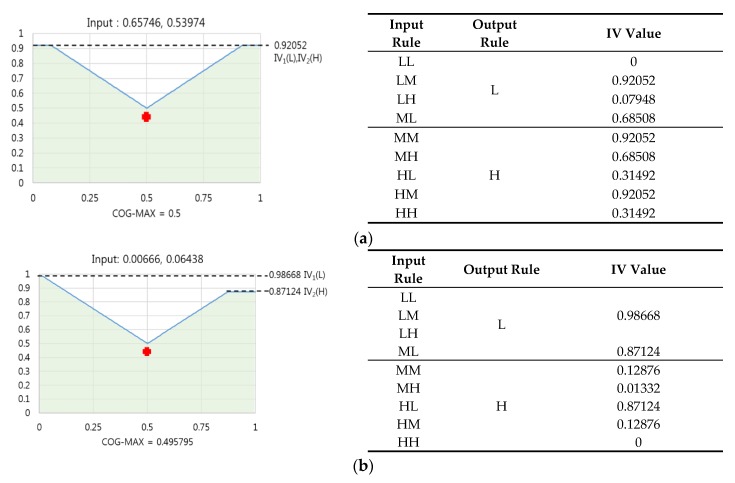

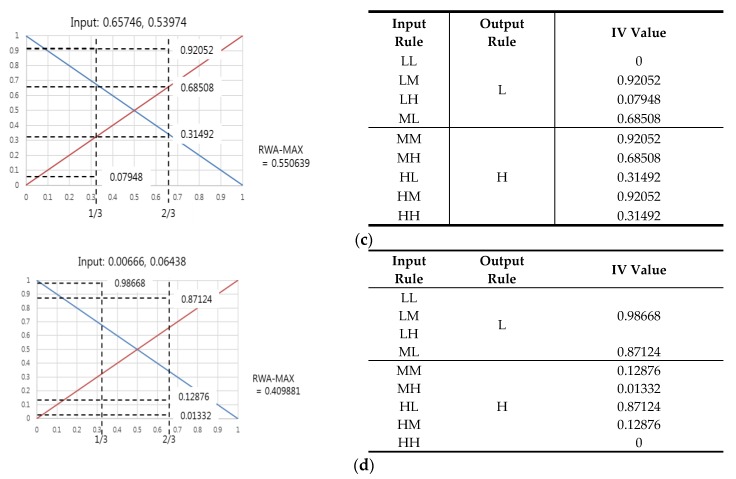
Comparison examples of COG and RWA output values: (**a**) COG output value from fit banknote data and the IV table used; (**b**) COG output value from unfit banknote data and IV table used; (**c**) RWA output value from fit banknote data and IV table used; (**d**) RWA output value from unfit banknote data and IV table used.

However, in case of RWA, all IVs were used, and each IV was multiplied by a corresponding weight before the mean was found. Thus, the values were more effectively distributed than in case of COG. For example, in the case of fit banknote data where the input values (VR mean, NIRT mean) were (0.65746, 0.53974), and in the case of unfit banknote data where the values were (0.00666, 0.06438), if the IVs were selected using the Max rule, and COG or the RWA method was used to find values, the COG produced an output of 0.5 for the first input and 0.495795 for the second input, as shown in Figure 9. On the contrary, RWA produced an output of 0.550639 for the first input and 0.409881 for the second input.

As we can see, in case of COG, the output values for fit and unfit banknote data were 0.5 and 0.495795, respectively, which are similar, whereas in the case of RWA, the outputs for fit and unfit banknote data were 0.550639 and 0.409881, which has a greater difference; hence, the latter was accurate in classifying banknote data.

Table 5 shows a comparison of the fitness classification results of the proposed method and a method proposed in previous research [9]. To perform banknote fitness classification, the method proposed in [9] used acoustic energy patterns that occur when banknotes pass over rollers. In the relevant paper, 40 one-dollar banknotes were used to perform an experiment to test performance; thus, in this study, the degree of accuracy was compared by using one-dollar banknotes as well. As can be seen in Table 5, the performance of our proposed method was superior to that of the previous method.

A comparison experiment was also performed with another previously proposed method that used a neural network [7]. Since the neural network requires training, a two-fold cross-validation method was employed, where the database used in this research was divided into two halves (T1 and T2). The EER was measured using T1 as the training set and T2 as the test set, and the EER was measured using T2 as the training set and T1 as the test set. The average EER was calculated to reflect the final performance. As shown in Table 6, the method proposed in this research (0.04%) yielded better performance than the other fitness classification method that used a neural network (3.32%). As shown in Table 6, the proposed method, unlike previous research [7], employs a fuzzy system that uses a suitable fuzzy rule in order to perform accurate banknote fitness classification.

**Figure 10:**
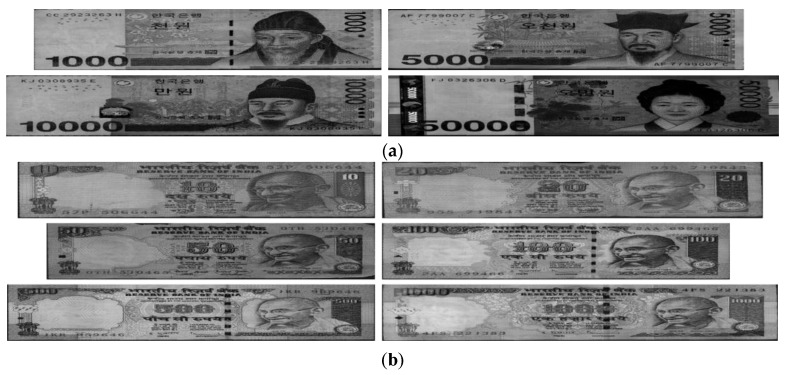
Examples of banknote images used in the experiment: (**a**) KRW; (**b**) INR.

In the next experiment, in order to check the performance of the proposed method on different types of banknotes, we used it on a database of images of banknotes of two other countries. The currencies used in the experiment were KRW and INR, and the number of banknote images and number of classes used in the experiment are shown in Table 7. Figure 10 shows examples of banknote images used for each national currency.

In the results of the experiments, a fitness classification mean error rate of 0.92% was achieved for KRW. In previous research [8], a discrete wavelet transform and support vector machine method had been used to perform fitness classification on INR. Thus, this method’s INR fitness classification accuracy was compared with that of the proposed method, as shown in Table 8. As the table shows, the proposed method yielded an EER of 0.06%, which was better than the results of the relevant research [8].

The previously mentioned performance by proposed method for the USD database (EER 0.04%), the KRW (EER 0.92%), and the INR (EER 0.06%) shows that the method has high fitness classification accuracy regardless of the type of database.

**Figure 11:**
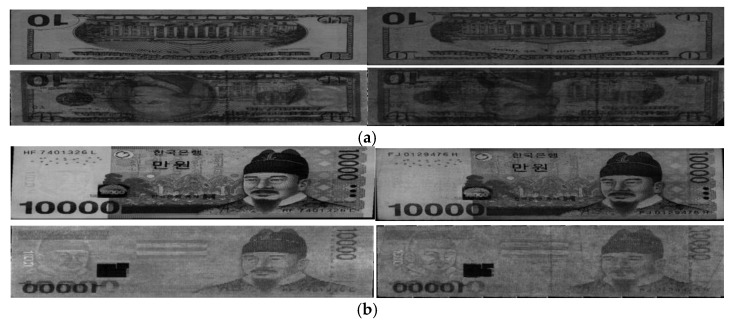

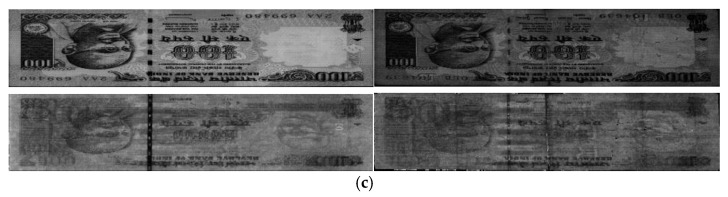
Examples of correctly recognized cases by our method on: (**a**) USD; (**b**) KRW; (**c**) INR. In (**a**–**c**), upper and lower figures represent the VR and NIRT images, respectively. In (a–c), left and right figures show the cases that fit and unfit banknotes are correctly recognized as fit and unfit ones, respectively.

In Figure 11, we show the examples of correctly recognized cases by our method on USD, KRW, and INR, respectively. As shown in Figure 11, we can confirm that our method can correctly discriminate the fit and unfit banknotes irrespective of various qualities of banknote images.

**Figure 12:**
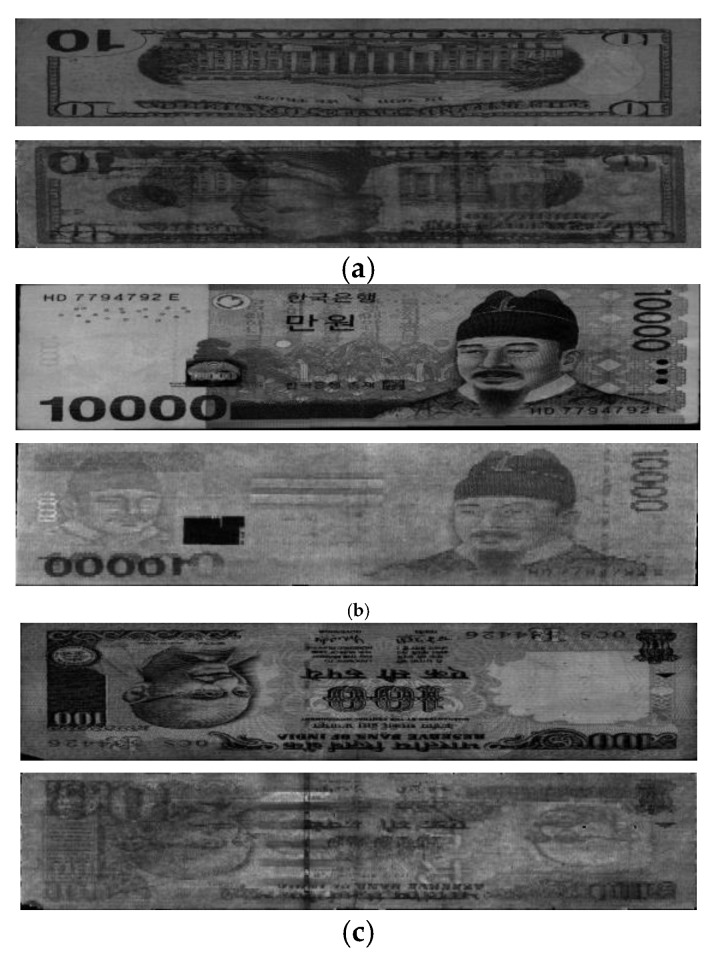
Examples of incorrectly recognized cases (Type I error) by our method on: (**a**) USD; (**b**) KRW; (**c**) INR. In (a–c), upper and lower figures represent the VR and NIRT images, respectively.

In Figure 12, we show the examples of incorrectly recognized cases (Type I error) by our method on USD, KRW, and INR, respectively. As shown in Figure 12, although they were manually classified as fit data based on densitometer measurement values [18], the average pixel values of ROI of VR and NIRT images are similar to those of unfit images, which causes the Type I error.

**Figure 13:**
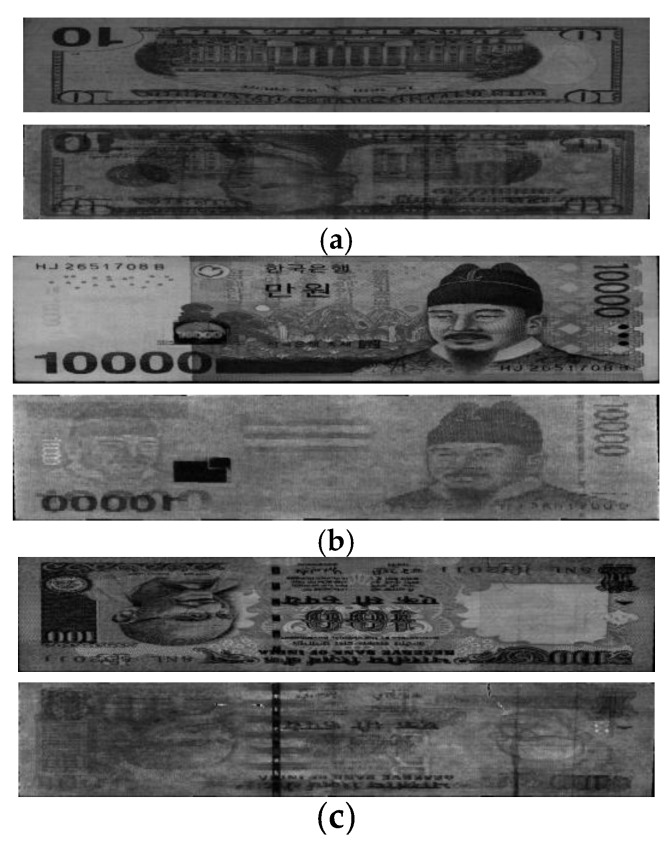
Examples of incorrectly recognized cases (Type II error) by our method on: (**a**) USD; (**b**) KRW; (**c**) INR. In (a–c), upper and lower figures represent the VR and NIRT images, respectively.

In Figure 13, we show the examples of incorrectly recognized cases (Type II error) by our method on USD, KRW, and INR, respectively. As shown in Figure 13, although they were manually classified as unfit data based on densitometer measurement values [18], the average pixel values of ROI of VR and NIRT images are similar to those of fit images, which causes the Type II error.

The processing time of proposed method was also measured. Measurement of processing time was performed on the desktop computer composed of an Intel(R) Core(TM) i7 Extreme 975 CPU of 3.33 GHz [19] and 8 GB RAM. In addition, the processing time was measured on the embedded system using a Texas Instruments (TI) digital media system-on-chip of 729 MHz (model name is TMS320DM6467 [20]) in an actual banknote counting machine. The processing time on the desktop computer was measured at an average of 0.1 ms per each image, and that the banknote counting machine was measured at an average of 1 ms per each image. All the time taken by all the procedures of our method (including the segmentation of banknote area and ROI detection, *etc*.) are included in the measured processing time. Because of this, we knew that the proposed method could perform fitness classification in a banknote counting machine at a rate of 1000 banknotes per second.

Finally, in order to use the proposed algorithm in banknote counting machines with limited resources, the total memory usage by our program was measured in the banknote counting machine. The measurement results, which are shown in Table 9, confirmed that the total memory usage was 931,231 bytes, and our program could run on the actual banknote counting machine without memory problems.

In addition, we make all our datasets of USD, KRW, and INR open through the website [21] so that other researchers can easily get access to our datasets. It is easy to obtain large banknote datasets for classifying the kinds of banknote such as $1, $10, and $100, *etc.* However, it is much difficult to obtain large banknote datasets for classifying the fitness and unfitness of banknote. That is because based on the actual densitometer measurement values [18] about soiling levels, human experts classified the banknotes in the database as fit banknotes (capable of being used) or unfit banknotes (incapable of use) for ground-truth data. Therefore, we performed the experiments with three datasets of USD (3856 images from 28 classes), KRW (3956 images from 16 classes), and INR (2300 images from 24 classes) as shown in Table 3, Table 4 and Table 7. Our experimental datasets are much larger than those of previous researches [7,8,9].

In our research, fuzzy-based classifier (based on heuristic design of fuzzy rules and membership functions) is used. Therefore, the possibility of overfitting with our experimental datasets can be less in our method than that by the methods requiring much amount of training procedure such as neural network and SVM, *etc.* As shown in the comparative results of Table 5, Table 6 and Table 8, our method outperforms previous researches based on neural network [7], SVM [8], and dynamic time warping (DTW) [9], which shows that the possibility of overfitting by our method is less than that by previous researches.

## 4. Conclusions

This study proposed a fitness classification method using VR and NIRT images of banknotes, and features were extracted from ROI that were then used as input in a fuzzy system. Experiment results confirmed that the proposed method yielded very accurate classification results for USD banknotes and other national currencies. In the future, we plan to test the proposed method using more currencies, such as the Euro and Chinese banknotes. Further, we plan to research a method with more than two classes of fitness classification, such as three classes or more.

## Figures and Tables

**Table 1:** Fuzzy rule table.

Input		Output
VR Mean	NIRT Mean	
L	L	L
	M	
	H	
M	L	
	M	H
	H	
H	L	
	M	
	H	

**Table 2:** Example of IVs obtained by Fuzzy rule, Min rule, or Max rule.

Index	Output by VR Mean	Output by NIRT Mean	Min Rule	Max Rule
1	0 (L)	0 (L)	0 (L)	0 (L)
2		0.8 (M)		0.8 (L)
3		0.2 (H)		0.2 (L)
4	0.4 (M)	0 (L)		0.4 (L)
5		0.8 (M)	0.4 (H)	0.8(H)
6		0.2 (H)		0.4 (H)
7	0.6 (H)	0 (L)	0 (H)	0.6 (H)
8		0.8 (M)	0.6 (H)	0.8 (H)
9		0.2 (H)		0.6 (H)

**Table 3:** Total number of images in the US dollar database used in experiments.

	Direction	A	B	C	D
Denomination					
$1		148			
$2		20			
$5		88			
$10		136			
$20		160			
$50		212			
$100		200			

**Table 4:** Number of fit and unfit images of US dollar database.

Denomination	Direction	Fit	Unfit
$1	A	80	68
	B		
	C	76	72
	D	63	85
$2	A	12	8
	B		
	C		
	D		
$5	A	60	28
	B	52	36
	C	68	20
	D	64	24
$10	A	76	60
	B	84	52
	C	96	40
	D	98	38
$20	A	8	152
	B		
	C		
	D		
$50	A	144	68
	B	130	82
	C	120	92
	D	124	88
$100	A	172	28
	B		
	C		
	D		

**Table 5:** Comparison of mean EER between the proposed method and a previous method. (unit: %)

Denomination	Fuzzy System	Previous Method [9]
$1	0	9.8

**Table 6:** Comparison of mean EER between the proposed method and a previous method. (unit: %).

Denomination (Direction)		Previous Research [7]			Proposed Method
		Train (T1) → Test (T2)	Train (T2) → Test (T1)	Average EER	
$1	A	0	0		0
	B		9.09	4.76	
	C		1.63	1.31	
	D	9.67	8.99	9.13	
$2	A	0	0	0	
	B				
	C				
	D				
$5	A	22.22	13.33	20.6	
	B	12.9	0	7.69	
	C	0		0	
	D				
$10	A	12.64		11.22	
	B	14.53		12.68	
	C	0		0	
	D		1.47	0.85	1.02
$20	A		0		0
	B				
	C				
	D				
$50	A	5.55	10.67	9.91	
	B	6.15	2.04	3.5	
	C	0	6.6	3.33	
	D		10.43	5.76	
$100	A		0		
	B				
	C		4.65	2.32	
	D		0		
Mean		2.98	2.46	3.32	0.04

**Table 7:** Number of images and classes for KRW and INR used in experiments.

Currency	Number of Images	Number of Classes
KRW	3956	16
INR	2300	24

**Table 8:** Comparison of the EER in INR fitness classification between the proposed method and a previous method. (unit: %).

Currency	Proposed Method	Previous Method [8]
INR	0.06	0.37

**Table 9:** Memory usage of the proposed algorithm in a banknote counting machine. (unit: bytes).

Item	Memory Usage
Input image	831,600 (1584 × 525 * pixels)
Banknote region	96,000 (400 × 120 pixels × 2 **)
ROI image	3600 (60 × 30 pixels × 2 **)
Proposed algorithm	31
Total	931,231

* 350 and 175 pixel are respectively for VR and NIRT images because the sizes of VR and NIRT images are 1584 × 350 pixels and 1584 × 175 pixels, respectively; ** VR and NIRT images.
